# Adaptive dynamics of unstable cancer populations: The canonical equation

**DOI:** 10.1111/eva.12625

**Published:** 2018-04-17

**Authors:** Guim Aguadé‐Gorgorió, Ricard Solé

**Affiliations:** ^1^ ICREA‐Complex Systems Lab Universitat Pompeu Fabra Barcelona Spain; ^2^ Institut de Biologia Evolutiva (CSIC‐UPF) Barcelona Spain; ^3^ Santa Fe Institute Santa Fe NM USA

**Keywords:** cancer adaptation, critical points, genome instability, Moran process, unstable dynamics

## Abstract

In most instances of tumour development, genetic instability plays a role in allowing cancer cell populations to respond to selection barriers, such as physical constraints or immune responses, and rapidly adapt to an always changing environment. Modelling instability is a nontrivial task, since by definition evolving instability leads to changes in the underlying landscape. In this article, we explore mathematically a simple version of unstable tumour progression using the formalism of adaptive dynamics (AD) where selection and mutation are explicitly coupled. Using a set of basic fitness landscapes, the so‐called canonical equation for the evolution of genetic instability on a minimal scenario associated with a population of unstable cells is derived. We obtain explicit expressions for the evolution of mutation probabilities, and the implications of the model on further experimental studies and potential mutagenic therapies are discussed.

## 1 INTRODUCTION

Cancer can be understood as the failure of those regulatory mechanisms that guarantee the maintenance of tissue and organ homoeostasis. Cooperative interactions along with extensive feedback signalling loops and replication checkpoints provide multiple paths to avoid the emergence of undesirable mutations or chromosomal abnormalities that can allow rogue cells to start proliferative growth. In dynamical terms, what has to be avoided within multicellular organisms is any kind of individual cell Darwinian evolution (Gatenby & Brown, 2017; Greaves & Maley, 2012; Nowell, 1976).

It is generally acknowledged that genetic instability plays a key role in tumour progression and carcinogenesis (Hanahan & Weinberg, 2011). Unstable genomes result from the failure of molecular mechanisms responsible for the maintenance of genome integrity (Negrini, Gorgoulis, & Halazonetis, 2010). That cancer cells are unstable is fairly well illustrated by the observation of their karyotypes: in sharp contrast with healthy cells, cancer chromosomal arrangements reveal a wide degree of aneuploidy (Lengauer, Kinzler, & Vogelstein, 1998). Such high levels of mutational load deploy the potential to overcome selection barriers, as well as involve a rather uncommon process from multicellularity to reduced cellular complexity (Solé et al., 2014), giving place to a highly adaptive and heterogeneous population. Genetic instability acts as a driver as well as the search engine for disease progression. An important (and not always appreciated) consequence of instability is that, once unleashed, it can easily grow as the lack of proper repair can damage other components of the check‐and‐repair cellular network.

Despite increasing knowledge of the molecular basis of unstable tumorigenesis, there is still the need for understanding the role of instability on cancer evolution, namely discerning if it is a cause or a consequence of carcinogenesis, how does it evolve along tumour development, and what are the treatment strategies that arise from answering such questions. Many mathematical models have provided interesting points into this topic, with the introduction of relevant ideas such as the mutator phenotype (Loeb, 2001) and several multi‐step models of mutation acquisition (see e.g., Komarova et al., 2002; Nowak et al., 2002) that have investigated the possible scenarios of correlation between instability and cancer progression.

The fact that genetic instability itself changes over cancer evolution makes it difficult to properly model its behaviour. Particular efforts, such as the computational models of Komarova, Sadovsky, & Wan, 2008 and Datta et al., 2013; have given interesting insight into understanding how a changing instability level affects, by means of modifying the probability of mutations, all kinds of replication and control mechanisms within the vast pathways towards cancer malignancy. Within this picture, instability cannot be taken as a parameter, but rather as an evolving phenotypic trait affected by the selective pressures of the tumour microenvironment. In this scope, the recent work by Asatryan and Komarova represents a further step for its proposal of an analytical approach where both instability and heterogeneity of cancer populations can be traced along time (Asatryan & Komarova, 2016). As a complementary point of view, we consider the need to include stochasticity in the process of acquiring either advantageous or deleterious mutations, together with considering instability as a trait evolving through changes within each single cell, compared to the idea of measuring it by following the competition dynamics between subpopulations with fixed mutation probabilities.

Here, we propose that the mean evolutionary paths of such stochastic process followed by unstable populations are describable by means of the framework of adaptive dynamics (AD) (Champagnat, Ferriere, & Ben Arous, 2001; Dieckmann & Law, 1996), which has been used in the study of cancer when focusing on niche construction (Gerlee & Anderson, 2015). AD models provide a powerful alternative to previous formal approaches by explicitly including replication, mutation and selection in a consistent way, allowing an exploration of the evolutionary dynamics of adaptive traits, while at the same time keeping a minimal, treatable model able to produce explicit expressions for trait evolution depending on a few parameters.

A central object in the AD framework is the so‐called canonical equation. For a given quantitative phenotypic trait *s*, this equation describes the evolutionary trajectory for the mean trait value as

(1) $$\frac{d\langle s\rangle }{dt}=\frac{1}{2}\mu \left(\left\langle s\right\rangle \right)\sigma^{2}\left(\left\langle s\right\rangle \right)n\left(\left\langle s\right\rangle \right){\left(\frac{\partial r(s,s^{\prime })}{\partial s^{\prime }}\right)}_{s^{\prime }=\left\langle s\right\rangle }$$

where μ(⟨s⟩) is the probability under which mutant individuals are generated, $\sigma^{2}$ is the variance of the mutant distribution *s′* derived from an individual with trait *s*,* n* the stationary population size and the last term in the right‐hand side stands for the fitness gradient associated with the specific landscape at work. The standard formulation involves some strong assumptions on the mutation‐selection process, and we will therefore review the mathematical process in order to understand up to which point the framework is suitable for our problem.

In the AD models, and in the work presented here, evolution takes place within a constant population context, where mutants appear and invade in a stepwise process, leading to a formalism for evaluating the trajectories of evolving trait values. This picture of cancer dynamics stems from classic work on ecological competition (Gatenby, 1995) where tumour cells act as invaders that cause the disruption of the local (tissue) ecology. These simplified models reveal how a proper formulation of competition can yield useful predictions (Gatenby, 1991). In this context, although the constant population falls short to describe the behaviour of some tumour growth processes, it is a much needed first approximation. Moreover, it can also be appropriate when dealing with some in vitro experiments involving long‐term evolution of unstable cancer cell populations. We will go back to this at the end of the paper.

Understanding how instability becomes a driver of evolvability can give further insight about its role as a cancer hallmark, and might as well produce relevant steps towards contemplating genetic instability as potential target for treatment. Is it possible to formulate a canonical equation describing the time evolution of instability? The answer is affirmative and here we show how it can be obtained.

## 2 POPULATION DYNAMICS

**Figure 1:**
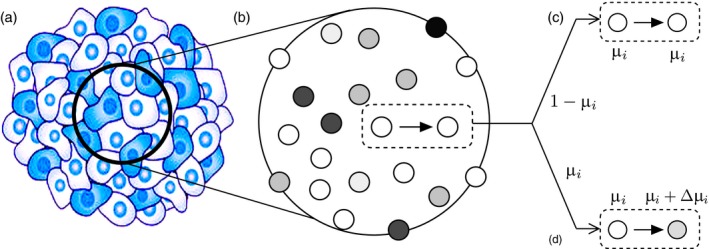
The Moran process rules associated with the model of a population of unstable cells competing for resources. We consider an idealized model of a heterogeneous cancer cell population (a) described by a well‐mixed (mean field) model (b). Here cells occupy a given domain that is not explicit and each cell has a distinct phenotype described in particular by its intrinsic instability μ_*i*_. In the Moran process, when a cell replicates it occupies another cell's niche and produces an identical daughter (c) or a slightly different one due to a mutation event proportional to μ_*i*_, which can lead to an increase $\Delta \mu_{i}$ of the instability levels (d)

With the aim of obtaining a clear understanding of the questions proposed above, we look for a minimal model to implement the unstable evolutionary dynamics. Our goal is to consider the process of cancer progression, which involves a heterogeneous population of cells (Figure 1a). In this population, cells are only characterized by their particular replication rate *r*_*i*_ and mutation probability μ_*i*_. However, and in the eyes of the AD approach, this preliminary model uses a constant population approximation where mutation probabilities are small enough so that the dynamics remain in equilibrium in‐between invasions. This approach, whose limitations will be later thoroughly discussed, is best described by means of a so‐called Moran process (Moran, 1958).

A particularity of the Moran process—here coarse‐grained into a continuous process, keeping in mind the long‐term evolution of tumour progression—is that cells of type *c*_*i*_ give birth by means of occupying other, randomly chosen cell sites at rate *r*_*i*_, so that the birth–death process is coupled into a single event (Figure 1c) that will eventually lead to selection towards cells with higher *r*_*i*_, thus producing a minimal environment where selection can take place. Furthermore, mutation is introduced by considering that cells can give birth to mutant offspring at probability μ_*i*_.

Mutations, however, do not occur as in quasispecies or replicator‐mutator models, where genomes mutate from one to another. In our model, a newly born mutant cell will have a modified mutation probability $\mu^{\prime }=\mu_{i}+\Delta \mu$, where Δμ is taken from a continuous distribution that we discuss later on. With this, we emphasize the wide levels of heterogeneity and genomic configurations found within tumours by means of giving a different phenotype to each cell rather than grouping populations into a countable, finite set of possible genome configurations.

Within this minimal model, we aim at understanding how selection and mutation are coupled when instability, and thus the individual mutation probability μ_*i*_, can itself change and affect the rate of cell replication *r*_*i*_, and what the evolutionary consequences of this coupling are.

## 3 SELECTION ON INSTABILITY

As discussed above, a most common event during the process of tumorigenesis are mutations in oncogenes that usually result in increased levels of replication (Vogelstein & Kinzler, 2002), thus giving to instability a role in activating the paths towards higher replicative capacity. On the other hand, the same elevated levels of instability can trigger deleterious mutations in genes that are vital for correct cellular metabolism and functioning, eventually leading to reduced cell viability or death. This apparent trade‐off supposes the existence of a clear coupling between replicative capacity, cell viability and mutation probability that sits at the basis of tumour replication, evolvability and adaptation. We hereby propose a minimal adaptive landscape that translates such coupling into replication rates being a function of instability, *r*(μ).

### 3.1 Adaptive landscape

**Figure 2:**
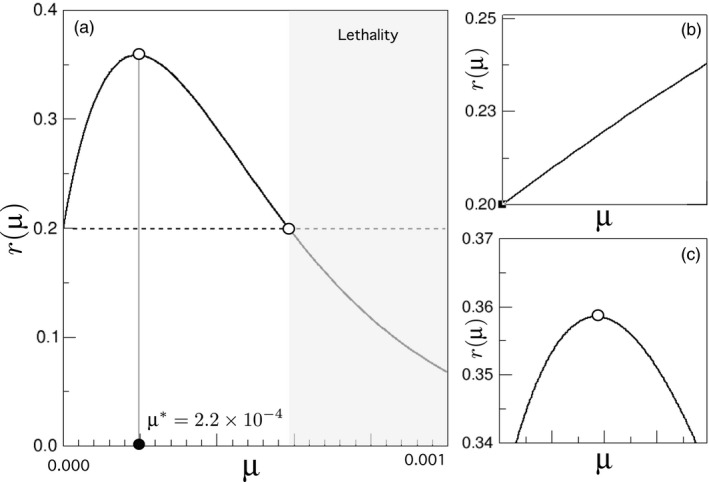
Fitness landscape function associated with the evolutionary dynamics of unstable tumour cells. In (a) the full landscape, given by a replication rate $r\left(\mu \right)=\left(r_{0}+N_{R}\delta_{R}\mu \right){\left(1-\mu \right)}^{N_{\mathrm{HK}}}$, is plotted against the instability probability μ. Further discussion is focused on two limit cases representing initial linear progression of instability (b) and optimal mutation (c) domains

Within our scope of producing a minimal model we expect to describe evolutionary dynamics on an adaptive landscape containing a reduced, treatable set of components. Taking into account the previously mentioned trade‐off, these follow from considering the effect of mutations enhancing malignant cell replication, provided that such mutations have not damaged any of the necessary machinery for cell viability. We start by considering that mutations on oncogenes can translate into a linear increase in replication rate, such that $r\left(\mu \right)=r_{0}+N_{R}\delta_{R}\mu$, with *r*_0_ being the basal replication rate of normal cells, *N*_*R*_ the number of oncogenes responsible for increased replication and $\delta_{R}$ the mean effect on replication rate when mutating one of such genes. Following a linear approximation, we do not include a saturation term for the number of nonmutated oncogenes. This is actually consistent with early stages of tumour evolution, where only a small fraction of oncogenes has been affected and so *N*_*R*_ can be kept as a constant. In this picture, we need as well to take into account the minimal genetic material needed for a cell to keep its basic functions. If we group such material into a number of house‐keeping genes, *N*_HK_, the probability that none of them has been mutated is ${\left(1-\mu \right)}^{N_{\mathrm{HK}}}$. Grouping both considerations together we obtain an analytical description of the coupling between replication and instability

(2) $$r\left(\mu \right)=\left(r_{0}+N_{R}\delta_{R}\mu \right){\left(1-\mu \right)}^{N_{\mathrm{HK}}}$$

(Solé et al., 2014). This adaptive landscape is of course of qualitative nature, and realistic fitness landscapes for unstable tumour environments are still far from our knowledge. However, certain points can be made if we give values within realistic parameter ranges to our function. The number of both oncogenes and house‐keeping genes have been widely assessed, and we take them to be about $N_{R}\approx 140$ (Vogelstein et al., 2013) and $N_{\mathrm{HK}}\approx 3804$ (Eisenberg & Levanon, 2013), respectively. Interestingly enough, considering small replication effects for $\delta_{R}$, such experimental values produce an adaptive landscape (Figure 2) that has a positive gradient within the region of $\mu \in [10^{-9},10^{-4}]$, so that our evolutionary trajectories will be bounded within a region of instability levels in accordance with those experimentally measured for tumour cells (Tomlinson, Novelli, & Bodmer, 1996).

### 3.2 Distribution of new mutations

We have assessed so far what is the effect of instability in proliferation, thus coupling mutation and selection for mutation level. Up next, we need to evaluate how does instability change during reproduction, so that we can finally compute the effects on replicative capacity of a mutated cell. As previously discussed, a broad range of mechanisms relates to variations in DNA replication fidelity. Such variations, however, are hardly in the direction of increasing DNA stability, and in general account for an increase in the mutation probability of cancer cells due to accumulation of further tumour‐suppressor or care‐taker gene mutations (Vogelstein & Kinzler, 2002).

This trend of generating more unstable offspring is translated into a positively skewed distribution of mutants M(μ,Δμ). To keep the mathematical background of our model treatable, a Rayleigh distribution peaked at Δμ=0 has been chosen^1^ . Under this scheme, instability of a daughter cell is likely to be similar or slightly higher from its parent, controlled by a scale parameter $\sigma_{\mu }^{2}$ depicting the general size of mutational increases.

## 4 ADAPTIVE DYNAMICS

Adaptive dynamics is a set of techniques or a mathematical framework that models long‐term phenotypic evolution of populations. Several works by different authors cover a broad scope of possible applications, and we hereby focus on the work of Dieckmann and Law and others (Champagnat et al., 2001; Dieckmann & Law, 1996) and adapt it to our particular system. The main biological background behind the maths sits in considering the evolutionary step as a mutant appearing and invading in a population in ecological or dynamical equilibrium (Dieckmann & Law, 1996). Under this picture, the ecological and evolutionary time scales are considered to be uncoupled, so that the process of the mutant competing against the resident population, and eventually fixating in it, is considered instantaneous in the evolutionary process.

General AD literature (see e.g., Champagnat et al., 2001; Dieckmann & Law, 1996; Geritz et al., 1998) follows the evolution of a quantitative phenotypic trait or set of traits, *s*, that can change through mutations. In these studies, the probability μ at which mutations appear is considered a possible function of the trait *s*, but afterwards and further on in the AD literature is usually left as a constant of each model. In the light of what we have discussed in the previous section, however, instability itself is a quantitative trait if computed as a mutation probability, and so the coupling of mutation and selection results in s  =  μ being the studied trait value.

The starting point of the AD modelling is to consider the evolutionary process, where the population's mutation probability changes as mutants appear and fixate, as a Markov chain for the probability of finding the population at time *t* having trait value μ

(3) $$\frac{dP(\mu ,t)}{\mathrm{dt}}=\int \left(w\left(\mu |\mu^{\prime }\right)P\left(\mu^{\prime },t\right)-w\left(\mu^{\prime }|\mu \right)P\left(\mu ,t\right)\right)d\mu^{\prime }.$$

The transition probabilities $w(\mu^{\prime }|\mu )$ describe the evolutionary step and contain the probability of the mutant with trait μ′ appearing (*A*) and fixating (ρ) in the population, so that $w\left(\mu^{\prime }|\mu \right)=A\left(\mu ,\mu^{\prime }\right)\rho \left(\mu ,\mu^{\prime }\right)$. The probability that a mutant appears is $A\left(\mu ,\mu^{\prime }\right)=Nr\left(\mu \right)\mu M\left(\mu ,\mu^{\prime }\right)$ , the size of the population at equilibrium *N*, the probability of birth and mutation r(μ)μ and the probability that the mutant has mutation probability μ′ provided the parent cell had probability μ.

The probability $\rho (\mu ,\mu^{\prime })$ that a mutant with fitness advantage $r\left(\mu^{\prime }\right)/r\left(\mu \right)$ fixates in a population of *N* individuals has an analytical expression for the Moran model (Ewens, 2004)

(4) $$\rho \left(\mu ,\mu^{\prime }\right)=\frac{1-(r\left(\mu \right)/r\left(\mu^{\prime }\right))}{1-{\left(r\left(\mu \right)/r\left(\mu^{\prime }\right)\right)}^{N}}.$$

A common procedure of the AD framework is to expand ρ for small variations of the trait value under the assumption of large populations, assumptions that are not a restriction for our problem. Under this view, the probability that the μ′ mutant fixates is zero for $r\left(\mu^{\prime }\right)\leq r\left(\mu \right)$ and

(5) $$\rho \left(\mu ,\mu^{\prime }\right)=\frac{\mu^{\prime }-\mu }{r(\mu )}{\left(\frac{\partial r}{\partial \mu^{\prime }}\right)}_{\mu^{\prime }=\mu }+O\left(\Delta \mu^{2}\right),$$

for $r\left(\mu^{\prime }\right)>r\left(\mu \right)$. Once a complete expression for the transition probabilities is build, we only need to recall how the evolution of the mean mutation probability can be written as

(6) $$\frac{d}{dt}\left\langle \mu \right\rangle \left(t\right)=\int \mu \frac{d}{dt}P\left(\mu ,t\right)d\mu ,$$

so that, using the original master equation, we obtain

(7) $$\frac{d}{dt}\left\langle \mu \right\rangle \left(t\right)=\int \int \left(\mu^{\prime }-\mu \right)w\left(\mu^{\prime }|\mu \right)P\left(\mu ,t\right)d\mu^{\prime }d\mu =\left\langle a_{1}\left(\mu \right)\right\rangle ,$$

where one recalls that $a_{k}\left(\mu \right)=\int {\left(\mu^{\prime }-\mu \right)}^{k}w\left(\mu^{\prime }|\mu \right)d\mu^{\prime }$ is the *k*‐th jump moment. If the first jump moment were a linear function of μ, then $\left\langle a_{1}\left(\mu \right)\right\rangle =a_{1}\left(\left\langle \mu \right\rangle \right)$ and the previous expression becomes directly treatable.^2^

The evolutionary trajectory for the mean path (we cease denoting it by angle brackets) will therefore follow

(8) $$\frac{d}{dt}\mu \left(t\right)=N\mu \frac{\partial r}{\partial \mu }\int {\left(\mu^{\prime }-\mu \right)}^{2}M\left(\mu ,\mu^{\prime }\right)d\mu^{\prime }$$

At this point, we recall that only fitter mutants can invade and so may eventually contribute to the exploration of the adaptive landscape. This translates onto the domain of the integration being restricted to $\mu^{\prime }>\mu$ , and so we integrate the positive part of our $\sigma_{\mu }^{2}$ skewed mutant distribution.

These considerations of selection on instability and nonsymmetrical mutations result in our first‐order approximation of the evolution of instability for a minimal cancer cell population:

(9) $$\frac{d\mu }{dt}=\gamma N\sigma_{\mu }^{2}\mu {\left(\frac{\partial r}{\partial \mu^{\prime }}\right)}_{\mu^{\prime }=\mu }$$

where

(10) $$\gamma =\frac{3}{\sqrt{e}}-\sqrt{\frac{\pi }{2}}\mathrm{erfc}\left(\frac{1}{\sqrt{2}}\right)$$

is simply a positive constant that results from integrating the asymmetric Rayleigh mutations distribution. Equation (9) defines the canonical equation for unstable cancer dynamics, describing the evolution of the mean mutation probability depending on population size, probability and effect of mutations and the steepness of the adaptive landscape defined by the effects of instability on cellular replication and viability.

## 5 EVOLUTION OF INSTABILITY

The canonical Equation (9) describes the evolution of instability in our model population depending on the population size *N*, the distribution of mutation jumps $\sigma_{\mu }^{2}$ and the product of the mutation probability and the gradient of the adaptive landscape $\mu \partial_{\mu }r$ . For our model landscape (Equation (5)), this turns out to be

(11) $$\begin{array}{cc} \frac{d\mu }{dt} & =\gamma N\sigma_{\mu }^{2}\mu \left(N_{R}\delta_{R}{\left(1-\mu \right)}^{N_{\mathrm{HK}}}\right.\\ & \left.-N_{\mathrm{HK}}\left(r_{0}+N_{R}\delta_{R}\mu \right){\left(1-\mu \right)}^{N_{\mathrm{HK}}-1}\right) \end{array}$$

**Figure 3:**
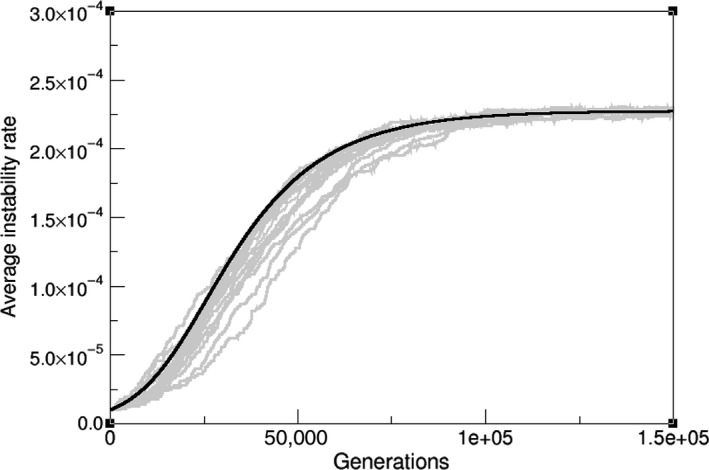
Evolutionary trajectories of the simulated Moran process (grey lines) and numerical solution of the predicted AD result (black curve), (population [*N*] = 2,000, distribution scale parameter [effect of mutations, σ] = 0.01, initial instability (μ_0_) = 10^−5^)

Complicated analytical solutions for this equation might not give best insight of the underlying dynamics. However, as a first test of our model we compare its numerical solution to averaged Moran Process simulations (Figure 3). It is both relevant and useful to understand the factors that cause deviations between computer experiments and our analytical approach, in order to further comprehend the approximations on which AD is build.

In terms of parameter range, these are mostly translated into the population being large enough, and mutation probabilities being proportionally small. The second is easily met for both healthy and cancerous human cells, but simulating full‐size clinically detectable tumours (more than 10^8^ cells (Bozic et al., 2013)) is of large computational cost, and keeping our model and exercise minimal, we have used smaller populations, modelling smaller subclones or spatially segregated populations where drift comes into play. Such drift produces a nonmonomorphic population where evolution deviates from the gradient trajectory and so proceeds slightly slower than our estimate. As previously stated and discussed along with the Appendix, the high nonlinearity of our landscape ensures that $\left\langle a_{1}\left(\mu \right)\right\rangle =a_{1}\left(\left\langle \mu \right\rangle \right)$ will be only valid up to a certain degree of approximation. It can be seen from Figure 3 that, still within this restricted range of validity, the canonical equation can capture the dynamics of instability up to a reasonable point.

A better understanding of the underlying dynamics can result from dividing the exploration of the landscape in well‐behaved regions where simpler equations will arise.

On the one hand, in an initial phase of malignancy exploration for small values of μ, the shape of the adaptive landscape is dominated by the linear increase of mutated oncogenes, $r\left(\mu \right)=r_{0}+N_{R}\delta_{R}\mu$ . Within this region, dynamics of instability follow

(12) $$\frac{d\mu }{dt}=\gamma N\sigma_{\mu }^{2}\mu N_{R}\delta_{R}$$

and the mean evolutionary trajectory is

(13) $$\mu \left(t\right)=\mu_{0}e^{\gamma \sigma_{\mu }^{2}NN_{R}\delta_{R}t}.$$

**Figure 4:**
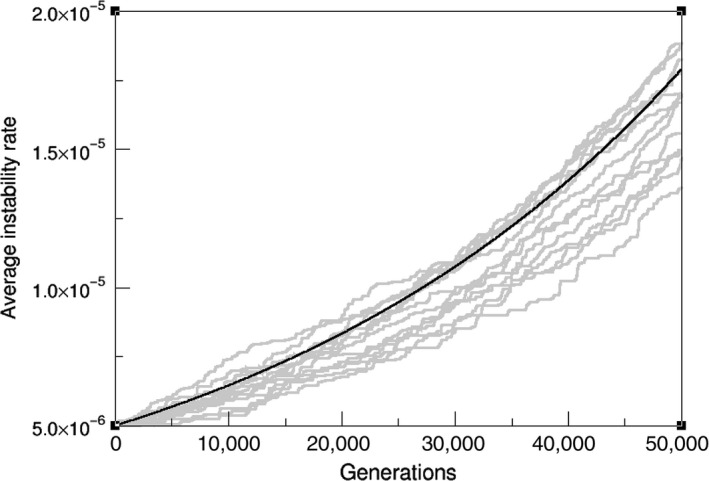
Exponential evolution of the mean mutation probability on a linear landscape: Moran process simulations (grey lines) of populations of 2,000 cells and the AD approximation (black curve), (σ = 0.01, μ_0_ = 5 × 10^−6^)

It is remarkable to understand how, even in a linear adaptive landscape, the coupling between mutation and selection on unstable cells introduces a further nonlinearity that will account for exponential exploration of the space of instability and the consequent exponential increases in replication capacity. Such results can be again compared to computer simulations of mutator‐replicator cells (Figure 4). The smaller nonlinearity also ensures that AD remains a good approximation despite stochastic deviation.

Another interesting point is to understand the behaviour of the mean instability levels as the population approaches the landscape peak. This kind of behaviour is easily studied if one considers a simple landscape containing a peak, such as $r\left(\mu \right)=r_{0}+\delta_{R}N_{R}\mu -\delta_{\mathrm{HK}}N_{\mathrm{HK}}\mu^{2}$ , where the role of house‐keeping genes is not considered totally deleterious but just reducing fitness quadratically with the mutation probability. This landscape has an optimal value at $\mu^{*}=\delta_{R}N_{R}/2\delta_{\mathrm{HK}}N_{\mathrm{HK}}$, and this peak is explored through

(14) $$\frac{d\mu }{dt}=\gamma N\sigma_{\mu }^{2}\mu \left(N_{R}\delta_{R}-2\delta_{\mathrm{HK}}N_{\mathrm{HK}}\mu \right).$$

**Figure 5:**
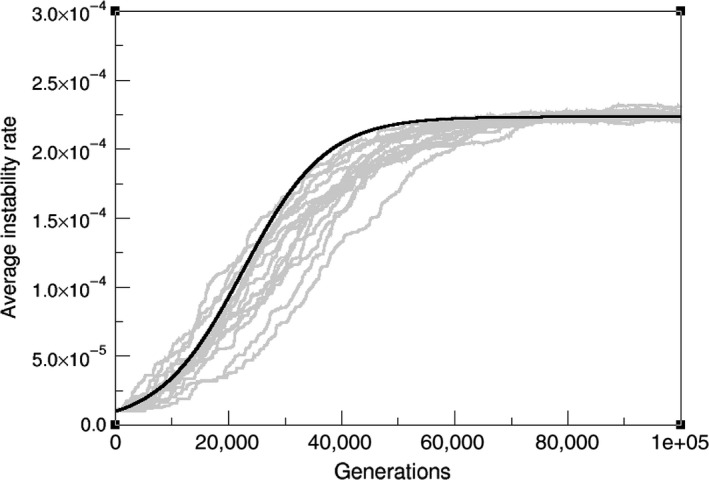
Mean mutation probability saturation at the fitness peak: Moran process simulations (grey lines) of populations of 2,000 cells and the AD approximation (black curve), (σ = 0.01, μ_0_ = 10^−5^)

By means of rewriting this trajectory as dμ/dt=Aμ(B−Cμ) , with $A=\gamma N\sigma_{\mu }^{2}$ , $B=N_{R}\delta_{R}$ and $C=2\delta_{\mathrm{HK}}N_{\mathrm{HK}}$ , its solution simplifies to

(15) $$\mu \left(t\right)=\frac{Be^{B(At+c_{1})}}{Ce^{B(At+c_{1})}+1},$$

where *c*_1_ ensures that μ(0)=μ_0_, the normal mutational probability of healthy cells. This trajectory saturates for long times at the expected result A/B=μ*, and can be again compared to computational experiments of replicating cells (Figure 5).

The same deviation between simulations and the numerical fit is found in this case, with evolution proceeding slower than our estimate. However, this minimal landscape approximation is able to capture the dynamical behaviour of our gene‐related landscape model, mainly with an initial exponential growth followed by saturation around the peak, which can be proven to be an evolutionary stable strategy (Geritz et al., 1998).

Provided that the canonical equation has a nontrivial, singular point, as we found for $\mu^{*}=\delta_{R}N_{R}/2\delta_{\mathrm{HK}}N_{\mathrm{HK}}$ , one can study the evolutionary stability of a quantitative trait. We can easily compute if this singular mutation probability will be an evolutionary trap, *that is*, a strategy that no further mutants can invade, if

(16) $${\frac{\partial^{2}r}{\partial \mu^{2}}}_{\mu =\mu *}<0$$

which holds for our strategy: $\partial_{\mu \mu }r\left(\mu \right)=-2\delta_{\mathrm{HK}}N_{\mathrm{HK}}<0$ .

## 6 DISCUSSION

In the present work, we have discussed the implications of the coupling between selection and instability for a minimal model of a population of mutating cells. We have shown how to determine the evolutionary trajectory for the mean instability levels in a basic landscape of cancer‐related genes. Our AD model, as defined by our canonical equation (and consistently with simulated trajectories), describes the tempo and mode at which mutation probabilities increase and saturate around fitness peaks. For a simple but sensible fitness landscape, a general canonical equation has been derived from the Moran process scenario. Several approximations have also been considered.

A first relevant result of our model arises from evaluating the canonical equation for unstable cells in a linear landscape, to be associated with a premalignant stage. The nonlinearity resulting from the coupling of mutation and selection predicts an exponential increase of instability levels, whereas a trait different from instability would only increase linearly within such landscape. This result is presented as a mathematical description of genomic instability being an enabling characteristic of cancer, by means of generating fast exploration of the space of possible mutations towards malignancy. Similarly, we obtained consistent matchings between simulated and average predicted instability values for the near‐optimum state. In this scenario, our model predicts an exponential increase followed by saturation around a critical mutational load, where, at least for this initial model of a nongrowing population, tumour cells are robust to further mutations. Considering that the distribution of mutational effects of cancer cells is hard to describe, it is important to understand that these results are qualitatively independent of the Rayleigh distribution, which we have only chosen in search for an asymmetric and analytically treatable function to work with. Other distributions would account for the same dynamics of exploration and saturation at different evolutionary paces. All in all, the possible applications of such minimal evolutionary descriptions of tumour instability follow from our set of examples and computer simulations.

Mounting evidence indicates that a successful approach to cancer therapy requires an explicit evolutionary perspective (Gatenby et al., 2009). One possible instance of this is provided by mutagenic therapies that have produced key results in the field of virology (Loeb et al., 1999). Would they be effective for cancer? Given some key analogies between RNA virus populations and unstable tumours (Solé & Deisboeck, 2004), this is an appealing possibility, although drug design or resistance mechanisms have yet to be assessed (Fox & Loeb, 2010). Prior to that, conceptual questions arise, such as do cancer cells live near critical instability levels, beyond which viability is no longer possible? is there a sharp error threshold for the mutation probability? what evolutionary outcomes should we expect when inducing variations on the mutational load of cancer cells, and how can these shed new light on mutagenic therapy?

Regarding the later, our model allows to bring instability as the evolving trait, while providing potential insights, particularly before and beyond the optimal instability levels. The exponentially fast increase of small mutational loads indicates that reducing instability levels in hope for progression delay might result in rapid re‐exploration of the mutator phenotype. On the other hand, pushing instability beyond optimal levels, even if a critical point is not trespassed (Solé & Deisboeck, 2004), might render tumour cells too unstable, and there exist relevant efforts towards using DNA repair inhibitors to produce critical instability levels (Helleday et al., 2008).

Our model differs from previous work in its simple analytical formulation, which do not depend on chosen parameter ranges, such as those of Datta et al., 2013 and Asatryan & Komarova, 2016; and they are thus qualitatively robust. On the one hand, this means that we are able to obtain analytical expressions for the exponential evolution and saturation of the mutation probability, which could eventually be used when studying *in vitro* long‐term evolutionary experiments with cancer populations, using serial transfer methods similar to those performed on viruses (see e.g., Drake, 1993; Sanjuán et al., 2010; Solé et al., 1999) or bacterial populations (see e.g., Moxon et al., 1994; Sniegowski, Gerrish, & Lenski, 1997; Barrick et al., 2009 for experiments and Taddei et al., 1997 for an early model for mutator alleles). Given the remarkable similarities found between microbial communities found both in the ecological and evolutionary time scales (Lambert et al., 2011), it would be worth exploring the evolution of instability of cancer cell cultures over many transfer generations (Langdon, 2004).

It remains an open question to analyse if any sort of saturating dynamics occur for both the fitness or the mutation probability when these experiments are performed on malignant cells. Furthermore, interesting theoretical approaches have been performed to infer the underlying adaptive landscape from the observable evolution of traits (Kryazhimskiy, Tkacik, & Plotkin, 2009). This seems a plausible point regarding how our model directly relates dynamics and landscape gradient and could therefore shed light onto understanding the evolutionary pressures underlying genetic instability at each stage of tumour progression.

On the other hand, while trying to produce a model that can be treated without the use of complex mathematical tools, we have been constrained to leaving aside many relevant considerations, the one we are most concerned with is the lack of growing population dynamics. This leaves our model interesting for the previously discussed specific confined experiments, while not yet complete when trying to study three‐dimensional growing tumours. While studying modifications to our formalism, following the work of evolutionary game theory on growing populations (see e.g., Li et al., 2015; Melbinger, Cremer, & Frey, 2010), we have decided to present this basic model as it remains a first step into a comprehensible and qualitative insight for the dynamics of populations able to evolve their mutation probability.

## CONFLICT OF INTEREST

None declared.

## Notes

1 The Rayleigh distribution is an asymmetric probability distribution defined for positive random variables (Forbes et al., 2010). We displaced so that its mode is zero, with shape

$$M\left(\Delta \mu ;\sigma_{\mu }^{2}\right)=\frac{\Delta \mu +\sigma_{\mu }}{\sigma_{\mu }^{2}}\text{exp}\left(\frac{-{\left(\Delta \mu +\sigma_{\mu }\right)}^{2}}{2\sigma_{\mu }^{2}}\right),$$

accounting for asymmetric probabilities of possible mutation levels, with small forward mutations being the most common.

2 It is interesting to understand the implications of such condition and how do they relate to the assumptions of the AD method and the limitations of our model. Between many approaches (see e.g., van Kampen, 1981; Kubo, Matsuo, & Kitahara, 1973), and without pretending to expose here a deep discussion on this aspect, one might consider extracting a Fokker–Planck equation from the Markov chain (6). By means of computing the evolution of the mean value $\left\langle \dot{\mu }\right\rangle$ for that equation, it can be seen that the dynamics of ⟨μ⟩ are only equivalent in both frameworks if either the selection gradient ∂r/∂μ does not depend on μ (a linear adaptive landscape), or the population is strictly monomorphic on the trait μ. A detailed explanation of this considerations can be found in the Appendix section. These conditions will impose a strong constraint when considering a realistic tumour environment, so that it will become necessary along our computational experiments and the discussion to assess the regions of validity of our model.

## APPENDIX 1

### Linearity of 〈*a*_1_(μ)〉

As discussed when introducing the AD approach, we have considered that the first jump moment is linear in μ to obtain the canonical equation. Understanding the mathematical basis behind such condition can give further insight into the assumptions our model sits on. Let us start again from a Master equation that describes the evolutionary biased random walk: the trait substitution sequence that we use to model evolution. This is

(17) $$\frac{dP(\mu ,t)}{dt}=\int \left(w\left(\mu |\mu^{\prime }\right)P\left(\mu^{\prime },t\right)-w\left(\mu^{\prime }|\mu \right)P\left(\mu ,t\right)\right)d\mu^{\prime }$$

We knew that *w*(μ, μ^′^), the transition probability, was the probability of a mutant appearing times the probability of a mutant surviving. If we consider the mentioned Moran process as a good evolutionary constant population model, the transition probabilities are those of our previous AD model

(18) $$w\left(\mu ,\Delta \mu \right)=N\left(\mu \right)r\left(\mu \right)\mu M\left(\mu ,\Delta \mu \right)\frac{\Delta \mu }{r(\mu )}\frac{\partial r}{\partial \mu }$$

and, supposing for simplicity that *M* is a symmetric distribution, we can obtain a Fokker–Planck transport equation of the form

(19) $$\frac{\partial p(\mu ,t)}{\partial t}=-\frac{1}{2}N\sigma_{\mu }^{2}\frac{\partial }{\partial \mu }\left(\mu \frac{\partial r}{\partial \mu }p\left(\mu ,t\right)\right)$$

The question is now: Under which assumptions the equation of the first moment of this distribution gives rise to the Canonical equation? Understanding these assumptions can give better insight into what are we actually doing with the canonical equation.

We can compute the dynamics of the first moment of the Fokker–Planck equation by means of multiplying by μ and integrating over the phase space, obtaining

(20) $$\frac{d\langle \mu \rangle }{dt}=\frac{1}{2}N\sigma_{\mu }^{2}\left\langle \mu \frac{\partial r}{\partial \mu }\right\rangle ,$$

which will only produce the Canonical equation if either the landscape is linear (and so 〈μ*r* ^′^〉 ∝ *k*〈μ〉) or the population is Dirac‐distributed (monomorphic), and so

(21) $$\left\langle \mu \frac{\partial r}{\partial \mu }\right\rangle =\int \mu \frac{\partial r}{\partial \mu }\delta \left(\mu -\left\langle \mu \right\rangle \right)d\mu =\mu \frac{\partial r}{\partial \mu }{|}_{\mu =\langle \mu \rangle }$$

This can serve as a basic mathematical description of why do populations have to be monomorphic in the AD framework for the canonical equation to arise, and why simulations with highly nonlinear landscapes deviate from our model approximation.
